# The Effect of Male Physicians’ Outfits on the General Population's Perception in Saudi Arabia

**DOI:** 10.7759/cureus.12131

**Published:** 2020-12-17

**Authors:** Ahmed Basheikh, Murad A Yasawy, Bashair M Magadmi, Yaroub Alganass, Almoutaz Hashim, Mazen Basheikh, Mohammed Basheikh

**Affiliations:** 1 Department of Ophthalmology, King Abdulaziz University, Jeddah, SAU; 2 Department of Medicine, Ibn Sina National College, Jeddah, SAU; 3 Department of Medicine, King Abdulaziz University, Jeddah, SAU; 4 Department of Internal Medicine, University of Jeddah, Jeddah, SAU

**Keywords:** physician, dress code, traditional, saudi arabia, white coat, western business suit, western tie, attire

## Abstract

Overview

Successful medical care depends on the trust developed between a physician and his patient. Professionally dressed doctors are likely to achieve a higher level of trust from their patients than those with a non-professional appearance.For many years, the physician’s famous white coat has been the standard professional wear around the world.

Few studies in Saudi Arabia have been conducted to analyze what kind of physician outfits patients prefer and whether the choice of attire affects the patient’s level of trust. These studies were either done in a single health institute, or in few primary healthcare clinics in one city.

This study aims to analyze whether the type of clothing worn by a physician improves the level of trust between a patient and a doctor. Participants were asked about different styles of clothing, including Western business attire, traditional Saudi outfits, and surgical scrubs, and whether wearing the white coat was preferred. Moreover, we sought to establish if differences in age, gender, nationality, or educational background affected the responses.

Methods

This cross-sectional study was carried out in August and September 2018. Participants living in Saudi Arabia anonymously filled out an electronic questionnaire, distributed by social media, which measured the effect of male physicians’ outfits on the general population's perception in Saudi Arabia. Participants were shown photographs of possible dress styles for physicians. Data were collected on the participants’ demographics, their most and least preferred doctor’s outfits, and the effect of the physician’s attire on their level of trust.

Results

A total of 8231 participants were included in the survey: 53% males, 87.9% with university-level education or higher, and 93.5% of Saudi nationality; 76.1% of the participants responded “yes” saying that the outfit would have an effect on how a patient might receive medical advice and follow the doctor’s recommendations. The most preferred outfits chosen by the participants were surgical scrubs with a white coat (39.3%), followed by a Western shirt and tie with a white coat (30.3%). The least preferred outfit was the full (traditional) Saudi outfit with a white coat (25.4%), followed by a Western business suit without a white coat (23.2%). The choices of most and least preferred attire were found to be impacted by different demographic factors, such as age, gender, and nationality, but not the educational background.

Conclusion

Physicians practicing in Saudi Arabia should note that their attire will earn the patient’s trust and encourage compliance with advice or treatment. The most preferred outfits were surgical scrubs with a white coat and a Western shirt and tie worn with a white coat, whereas the least preferred ones were the traditional Saudi thobe, with and without shemagh or ghutra, and the Western business suit without a white coat.

## Introduction

Establishing trust between a physician and his patients is a crucial component of medical care. Gaining this trust relies on many different factors, including the physician’s appearance [1]. Doctors will gain a patient’s trust if they have a good reputation and an intelligent manner of speaking, and professional attire is also important; a physician with a non-professional appearance will appear less trustworthy [2]. The importance of the physician’s dress code in establishing a trusting relationship between the patient and the physician can be traced back to Hippocrates [3].

For many years, the doctor’s standard attire around the world has been based on the famous white coat [4]. In Western countries, however, physicians are trying to gain more compliance from patients by dressing in casual attire to break down the doctor-patient barrier [5-7]. This is not the case in Arabic countries, such as Saudi Arabia, where the white coat is preferred, with recent growing popularity for surgical scrubs [8].

Few studies have been conducted in Saudi Arabia to address the effect of the physician’s attire on patient trust or what sort of standard attire is preferred by patients [8-10]. These studies were either carried out in a single health institute or in few primary healthcare clinics in one city.

This study measured the acceptance and preference of the Saudi population by presenting photographs of different styles, such as Western wear (shirt and tie or business suit), traditional Saudi dress thobe (long white robe) with or without a shemagh (red overhead scarf) or ghutrah (white overhead scarf), and finally surgical scrubs, all of these either with or without a white coat. We correlated the findings to the participants’ age, gender, nationality, and educational background.

## Materials and methods

This cross-sectional study was carried out in August and September 2018. Participants living in Saudi Arabia anonymously filled out an electronic questionnaire, distributed by social media, which measured the effect of male physicians' outfits on the general population's perception in Saudi Arabia. The questionnaire included photographs of a male doctor wearing eight different professional dress styles. Data collection included the participant’s demographics, educational background, preference of the favorite and least favorite outfits, and whether the outfit would earn the patient’s trust.

**Figure 1:**
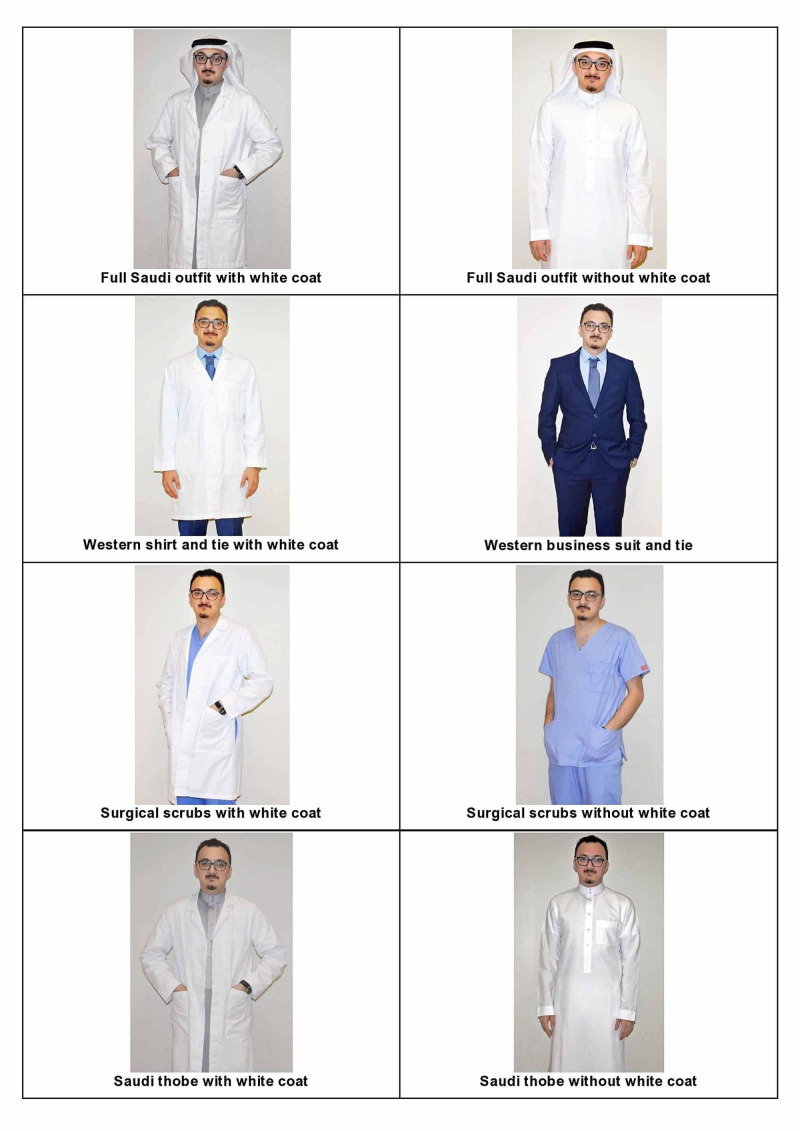
Different dress code choices included in the questionnaire

The model in the photographs was a volunteer member of the research team and provided expressed written consent to allow the publication of his photographs. The surveys included no personal information that would allow the participants to be identified. Confidentiality of data was ensured, and data was only accessed by the researchers. Questionnaires answered incompletely were excluded (Table 1 and Figure 1). Ethical approval for this study was obtained from the Biomedical Ethics Research Committee at King Abdulaziz University, Jeddah, Saudi Arabia.

**Table 1:** Questionnaire given to participants

Question	Response
Do you think that the outfit worn by doctors has any effect on how well you would accept their professional advice and follow their recommendations?	- Yes
	- No
Which of the following dress codes (see the photos) would you MOST prefer to see the doctor wearing in outpatient clinics?	- Full Saudi outfit with white coat
	- Full Saudi outfit without white coat
	- Western shirt and tie with white coat
	- Western business suit and tie
	- Surgical scrubs with white coat
	- Surgical scrubs without white coat
	- Saudi thobe with white coat
	- Saudi thobe without white coat
Which of the following dress codes (see the photos) would you LEAST prefer to see the doctor wearing in the outpatient clinics?	- Full Saudi outfit with white coat
	- Full Saudi outfit without white coat
	- Western shirt and tie with white coat
	- Western business suit and tie
	- Surgical scrubs with white coat
	- Surgical scrubs without white coat
	- Saudi thobe with white coat
	- Saudi thobe without white coat

Statistical analysis was performed using IBM Statistical Package for the Social Sciences (SPSS) Statistics version 20.0 (IBM Corp., Armonk, NY) to evaluate and test the hypothesis. We generated simple/cross-tabulation frequency tables and percentages. A chi-square test was used to test and describe the relationship between two categorized variables. The level P < 0.05 was used as the cutoff value for significance.

## Results

The survey generated 8231 respondents: 35.7% were between 18 and 25 years old, 32.1% between 26 and 34 years old, and 27% between 35 and 54 years old. Fifty-three percent of the participants were males. Most of the participants were of Saudi nationality (93.5%). University degrees were held by 68.4% of respondents; 19.5% had a postgraduate degree, and 11% had a high school degree (Table 2).

**Table 2:** Participant demographics

Category	Frequency	Percent
Age		
Less than 18	149	1.8
18–25	2942	35.7
26–34	2643	32.1
35–54	2220	27.0
55–65	234	2.8
Greater than 65	43	0.5
Total	8231	100.0
Gender		
Female	3868	47.0
Male	4363	53.0
Total	8231	100.0
Nationality		
Saudi	7697	93.5
Non-Saudi	534	6.5
Total	8231	100.0
Educational level		
Elementary school	7	0.1
High School	917	11.1
University	5633	68.4
Postgraduate	1601	19.5
Not educated	6	0.1
Middle school	67	0.8
Total	8231	100.0

When asked whether the outfit worn by a doctor would change their perception of the advice received and likelihood to follow recommendations, 76.1% responded with “yes” and 23.9% with “no”. The percentage of those who responded with “yes” was slightly higher in males than females, but statistically significant (77.6% and 74.5% respectively, P = 0.001).

The most preferred outfit or combination of outfits chosen by the participants was surgical scrubs with a white coat (39.3%), followed by a Western shirt and tie worn with a white coat (30.3%). Full traditional Saudi wear (thobe, and shemagh or ghutra) with a white coat was chosen by 8.6% of the participants (Table 3).

**Table 3:** Overall preferences for outfits to be worn by doctors in outpatient clinics

Outfit	Frequency	Percent
Full Saudi outfit with white coat	710	8.6
Full Saudi outfit without white coat	471	5.7
Western shirt and tie with white coat	2496	30.3
Western business suit and tie	141	1.7
Surgical scrubs with white coat	3237	39.3
Surgical scrubs without white coat	672	8.2
Saudi thobe with white coat	437	5.3
Saudi thobe without white coat	67	0.8
Total	8231	100.0

**Figure 2:**
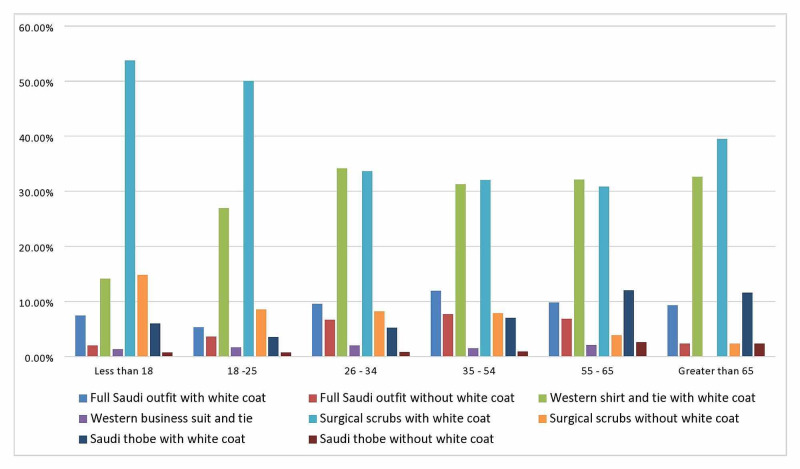
Preference for doctors’ outfits in relation to participant age groups (P < 0.0005)

Statistical analysis showed that the age of the participant significantly correlated with a choice. Almost 50% of the participants in the age group 18 or below or 18-25 preferred the combination of surgical scrubs with a white coat. That same choice was selected by a smaller percentage of older adults: 33.6% of the participants in the age group 26-34 and 32% in the age group 35-54 (P < 0.0005). See Table 4 and Figure 2 for more details.

**Table 4:** Outfit preferences in relation to participants’ age groups (P < 0.0005)

Age	Outfit preference								Total
	Full Saudi outfit with white coat	Full Saudi outfit without white coat	Western shirt and tie with white coat	Western business suit and tie	Surgical scrubs with white coat	Surgical scrubs without white coat	Saudi thobe with white coat	Saudi thobe without white coat	
Less than 18	11	3	21	2	80	22	9	1	149
	7.4%	2.0%	14.1%	1.3%	53.7%	14.8%	6.0%	0.7%	100.0%
18-25	156	106	792	46	1471	249	102	20	2942
	5.3%	3.6%	26.9%	1.6%	50.0%	8.5%	3.5%	0.7%	100.0%
26-34	252	175	901	54	887	217	137	20	2643
	9.5%	6.6%	34.1%	2.0%	33.6%	8.2%	5.2%	0.8%	100.0%
35-54	264	170	693	34	710	174	156	19	2220
	11.9%	7.7%	31.2%	1.5%	32.0%	7.8%	7.0%	0.9%	100.0%
55-65	23	16	75	5	72	9	28	6	234
	9.8%	6.8%	32.1%	2.1%	30.8%	3.8%	12.0%	2.6%	100.0%
Greater than 65	4	1	14	0	17	1	5	1	43
	9.3%	2.3%	32.6%	0.0%	39.5%	2.3%	11.6%	2.3%	100.0%
Total	710	471	2496	141	3237	672	437	67	8231
	8.6%	5.7%	30.3%	1.7%	39.3%	8.2%	5.3%	0.8%	100.0%

**Figure 3:**
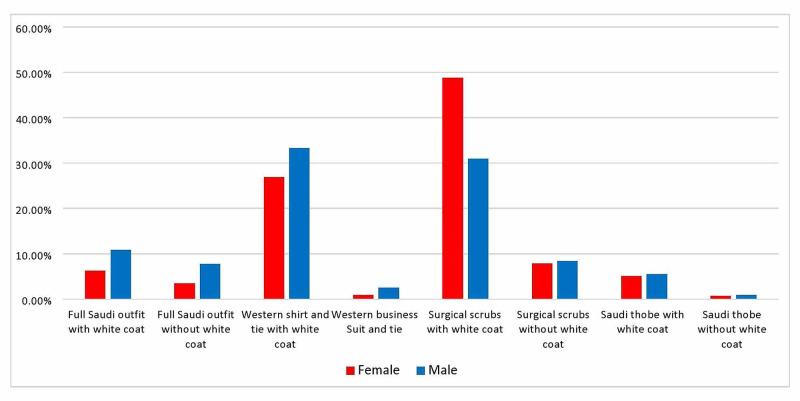
Outfit preference according to participant gender (P < 0.0005)

There is a significant correlation between the participants’ choices and gender: 48.8% of the females preferred the doctor wearing surgical scrubs with a white coat, and 26% chose a Western shirt and tie with a white coat (P < 0.0005). On the other hand, 33.3% of males preferred the doctor wearing a Western shirt and tie with a white coat, and 30.9% chose surgical scrubs with a white coat (Figure 3).

The comparison of outfit preference by nationality was also statistically significant. The most preferred option by Saudi participants was surgical scrubs with a white coat (39.4%), followed by a Western shirt and tie with a white coat (29.8%), and then a full Saudi outfit with a white coat (9%). Non-Saudi nationals preferred the Western shirt and tie with a white coat (38.4%), followed by surgical scrubs with a white coat (37.6%) and surgical scrubs without a white coat (11.6%) (P < 0.0005, Table 5).

**Table 5:** Outfit preference by participant nationality (P < 0.0005)

Nationality	Preferred outfit								Total
	Full Saudi outfit with white coat	Full Saudi outfit without white coat	Western shirt and tie with white coat	Western business suit and tie	Surgical scrubs with white coat	Surgical scrubs without white coat	Saudi thobe with white coat	Saudi thobe without white coat	
Saudi	696	463	2291	116	3036	610	421	64	7697
	9.0%	6.0%	29.8%	1.5%	39.4%	7.9%	5.5%	0.8%	100.0%
Non-Saudi	14	8	205	25	201	62	16	3	534
	2.6%	1.5%	38.4%	4.7%	37.6%	11.6%	3.0%	0.6%	100.0%
Total	710	471	2496	141	3237	672	437	67	8231
	8.6%	5.7%	30.3%	1.7%	39.3%	8.2%	5.3%	0.8%	100.0%

The level of education did not have a statistically significant correlation to the participants’ preferences.

Overall, the least preferred outfits chosen by the participants were the full Saudi outfit with a white coat (25.4%), followed by a Western business suit without a white coat (23.2%). Formal Saudi wear without a white coat was the least preferred by 11.9%, and the surgical scrubs alone without a white coat was chosen by 11.7% (Table 6).

**Table 6:** Least preferred outfits chosen by the participants

Outfit	Frequency	Percent
Full Saudi outfit with white coat	2088	25.4
Full Saudi outfit without white coat	976	11.9
Western shirt and tie with white coat	427	5.2
Western business suit and tie	1912	23.2
Surgical scrubs with white coat	533	6.5
Surgical scrubs without white coat	960	11.7
Saudi thobe with white coat	260	3.2
Saudi thobe without white coat	1075	13.1
Total	8231	100.0

**Figure 4:**
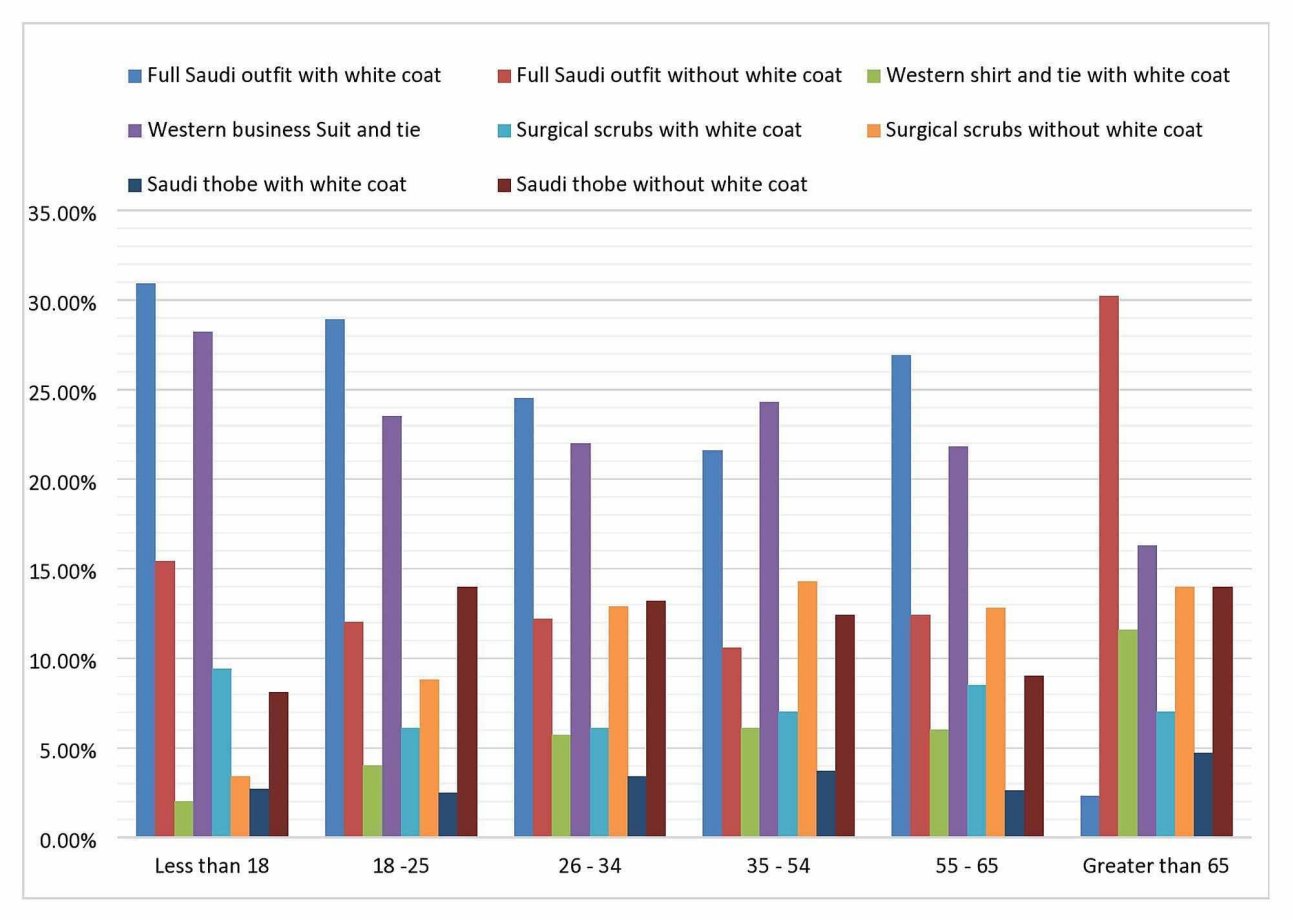
The least preferred outfits according to participants’ age groups (P < 0.0005)

The ages of the participants showed statistical significance when looking at the least preferred outfit (P < 0.0005). Full Saudi outfit with a white coat was the least preferred choice, at 30.9%, 28.9%, and 26.9% for the age group 18 or below, 18-25, and 35-54, respectively. A significant proportion of the participants older than 65 years (30.2%) did not like formal Saudi wear without a white coat (Figure 4).

**Figure 5:**
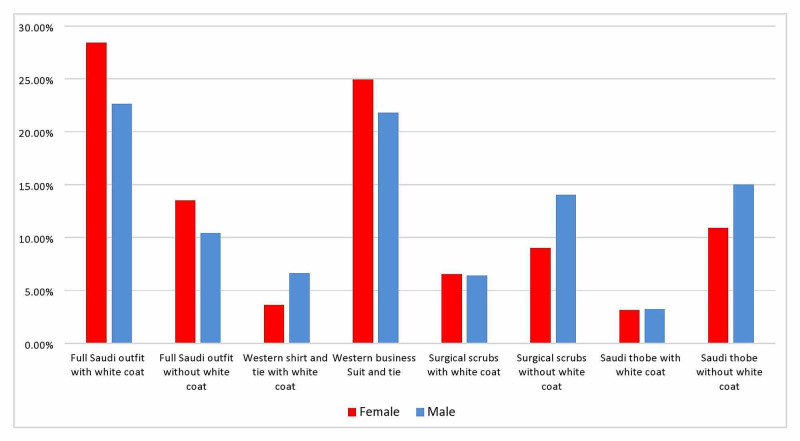
Least preferred outfits according to gender (P < 0.0005)

There was a statistically significant difference when looking at least favorite outfits according to the participants’ gender: females chose full Saudi wear with a white coat as least favorite (28.4%), followed by the Western business suit (24.9%), and full Saudi wear without a white coat (13.5%). Males chose the full Saudi outfit with a white coat (22.6%), followed by the Western business suit (21.8%), and thobe alone without a white coat (15%) (P < 0.0005, Figure 5).

When looking at the least preferred outfits by participant nationality, Saudi nationals chose the full Saudi outfit with a white coat (25.3%), followed by the Western business suit without a white coat (23.8%). Non-Saudi nationals chose the full Saudi outfit with a white coat as the least preferred doctor’s outfit (25.8%), followed by the thobe without a white coat (16.5%), and the full Saudi outfit without a white coat (15.4%) (P < 0.0005, Table 7).

**Table 7:** Least preferred doctor’s outfits by participant nationality (P < 0.0005)

Nationality	Least preferred wear								Total
	Full Saudi outfit with white coat	Full Saudi outfit without white coat	Western shirt and tie with white coat	Western business suit and tie	Surgical scrubs with white coat	Surgical scrubs without white coat	Saudi thobe with white coat	Saudi thobe without white coat	
Saudi	1950	894	388	1835	501	896	246	987	7697
	25.3%	11.6%	5.0%	23.8%	6.5%	11.6%	3.2%	12.8%	100.0%
Non-Saudi	138	82	39	77	32	64	14	88	534
	25.8%	15.4%	7.3%	14.4%	6.0%	12.0%	2.6%	16.5%	100.0%
Total	2088	976	427	1912	533	960	260	1075	8231
	25.4%	11.9%	5.2%	23.2%	6.5%	11.7%	3.2%	13.1%	100.0%

The level of education had no significant relationship to the participants’ choices of least preferred doctor’s outfit.

## Discussion

In this study, 76% of the participants responded with “yes” saying that the outfit worn by doctors had an effect on how the patients would accept medical advice and follow recommendations. This percentage corresponds with other studies done in Saudi Arabia and worldwide [1,9-11]. The percentage who perceives a physician’s appearance as important does not depend on the participant’s age or educational background.

In our study, the two most preferred male doctors’ outfits were surgical scrubs with a white coat (39.3%) and a Western shirt and tie worn with a white coat (30.3%). These findings compare favorably with other studies done in Saudi Arabia, where a Western shirt and tie with a white coat was the most preferred outfit [8-10]. The traditional Saudi thobe, with and without shemagh or ghutra, was not a popular choice when participants were asked to select the most preferred male doctor’s outfit, especially when worn with a white coat (the least preferred outfit chosen). This is similar to the results of the study done by Al-Ghobain et al., where only 9.7% of participants preferred traditional Saudi attire [8]. The other outfit less preferred by our study’s participants was the Western business suit without a white coat, which was selected by only 1.7% as the most preferred doctor’s outfit and was selected by 23.2% as the least preferred outfit. These findings are comparable with studies in the United States by Rehman et al. and Petrilli et al. [1,11].

The participants’ selections were affected by multiple factors, such as age, gender, and nationality. The younger age groups in this study (25 years old and younger) preferred surgical scrubs, with approximately 50% choosing surgical scrubs with a white coat and 10% choosing surgical scrubs without a white coat. This could be related to media effect and the publicity of the medical television shows, as these shows receive major acceptance among this age group; the stars of the shows are seen mostly wearing surgical scrubs, with and without a white coat. Of note, the least preferred doctor’s outfit by non-Saudi national participants was the choices that involved the Saudi traditional outfit. The educational background seems to have no influence over the participant choices.

The strength of the study comes from a large number of participants, allowing detailed analysis of the sample, and the questionnaire included respondents from all regions of the kingdom rather than one center or city as was done in previous studies. This large study allows a better understanding of patient preferences in Saudi Arabia.

This study had some limitations. First, it focused only on the attire of male doctors and did not address patient preferences for the outfits of female doctors, and another is the missing correlation to different doctors’ specialties and to different regions and cultural backgrounds of Saudi Arabia.

## Conclusions

Male physicians practicing in Saudi Arabia in outpatient settings should pay attention to what they wear as this affects the patient’s perception and compliance with advice or treatment. The most preferred outfits were surgical scrubs with a white coat and the Western shirt and tie worn with a white coat. The traditional Saudi thobe, with and without shemagh or ghutra, was the least preferred physician outfit, especially when worn with a white coat. The Western business suit with a tie was the second least favorite. The choices of most and least preferred attire were found to be impacted by different demographic factors, such as age, gender, and nationality, but not the educational background.
